# Impact of Hydrogen Sulfide on Mitochondrial and Bacterial Bioenergetics

**DOI:** 10.3390/ijms222312688

**Published:** 2021-11-24

**Authors:** Vitaliy B. Borisov, Elena Forte

**Affiliations:** 1 Belozersky Institute of Physico-Chemical Biology, Lomonosov Moscow State University, Leninskie Gory, 119991 Moscow, Russia; 2 Department of Biochemical Sciences, Sapienza University of Rome, 00185 Rome, Italy; elena.forte@uniroma1.it

**Keywords:** hydrogen sulfide, donors, gasotransmitters, molecular bioenergetics, inhibition, electron transport chain

## Abstract

This review focuses on the effects of hydrogen sulfide (H_2_S) on the unique bioenergetic molecular machines in mitochondria and bacteria—the protein complexes of electron transport chains and associated enzymes. H_2_S, along with nitric oxide and carbon monoxide, belongs to the class of endogenous gaseous signaling molecules. This compound plays critical roles in physiology and pathophysiology. Enzymes implicated in H_2_S metabolism and physiological actions are promising targets for novel pharmaceutical agents. The biological effects of H_2_S are biphasic, changing from cytoprotection to cytotoxicity through increasing the compound concentration. In mammals, H_2_S enhances the activity of F_o_F_1_-ATP (adenosine triphosphate) synthase and lactate dehydrogenase via their *S*-sulfhydration, thereby stimulating mitochondrial electron transport. H_2_S serves as an electron donor for the mitochondrial respiratory chain via sulfide quinone oxidoreductase and cytochrome *c* oxidase at low H_2_S levels. The latter enzyme is inhibited by high H_2_S concentrations, resulting in the reversible inhibition of electron transport and ATP production in mitochondria. In the branched respiratory chain of *Escherichia coli*, H_2_S inhibits the *bo*_3_ terminal oxidase but does not affect the alternative *bd*-type oxidases. Thus, in *E. coli* and presumably other bacteria, cytochrome *bd* permits respiration and cell growth in H_2_S-rich environments. A complete picture of the impact of H_2_S on bioenergetics is lacking, but this field is fast-moving, and active ongoing research on this topic will likely shed light on additional, yet unknown biological effects.

## 1. Introduction

**Figure 1:**
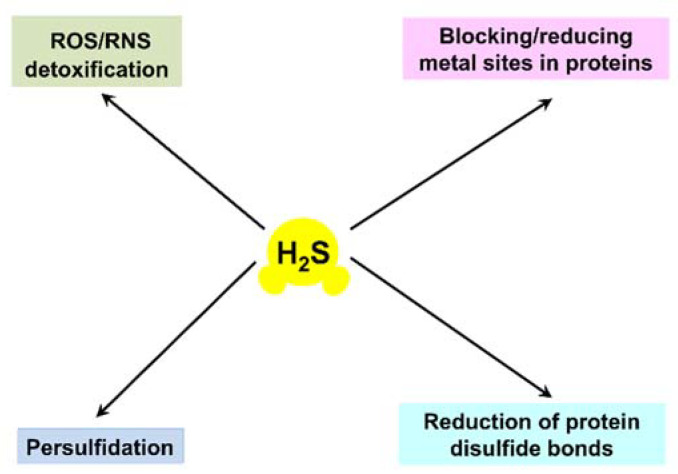
Proposed H_2_S signaling mechanisms in mammalian systems.

For a long time, hydrogen sulfide (H_2_S) had been considered merely as a highly toxic and occasionally lethal gas. However, it was discovered that in mammals H_2_S has physiological relevance and is endogenously generated [1,2]. Currently, H_2_S is considered to be a member of the class of gasotransmitters or, in other words, endogenous gaseous signaling molecules, along with nitric oxide and carbon monoxide [3,4,5,6,7]. Although still debated, cyanide has also recently been proposed to be part of this class [7,8]. H_2_S contributes to the regulation of important physiological processes in the cardiovascular, gastrointestinal, nervous, and respiratory systems. It shows various physiological effects in mammalian cells, but in a biphasic, concentration-dependent manner. At low, nanomolar concentrations, H_2_S exhibits cytoprotective effects. At higher levels the compound induces cytotoxicity. For instance, H_2_S exerts vasorelaxant effects through the opening of K_ATP_ (adenosine triphosphate) channels in vascular smooth muscle cells [9]. It also functions as a neuromodulator in the brain [1] and serves as a stimulator of angiogenesis [10]. At concentrations of 200 µM and higher, H_2_S induces apoptosis of aorta smooth muscle cells through the activation of mitogen-activated protein kinases and caspase-3 [11]. In mammalian systems, H_2_S is proposed to signal via four different mechanisms: (i) serving as an antioxidant that detoxifies reactive oxygen species (ROS) and/or reactive nitrogen species (RNS); (ii) blocking and/or reducing metal active sites in metalloproteins, e.g., heme Fe sites in heme proteins; (iii) performing protein *S*-persulfidation, also known as *S*-sulfhydration (post-translational modification of a cysteine residue by adding a thiol group); (iv) performing the chemical reduction of disulfide bonds in proteins (see [12,13] and references therein) (Figure 1).

The physiological roles of H_2_S are not limited to mammals but appear to be inherent in all kingdoms of life. In higher plants, H_2_S signaling functions seem to occur mainly through a persulfidation-based mechanism [14]. It regulates important physiological functions, including fruit ripening; stomatal movement; senescence in flowers, leaves, and fruits; photosynthesis via promotion of photosynthetic enzyme expression and chloroplast biogenesis; and the promotion of root organogenesis, seed germination, nodulation, and N_2_ fixation. H_2_S can also activate antioxidant systems in plant cells, thereby contributing to defense against adverse environment situations, such as drought-induced oxidative stress, salinity stress, temperature stress (high and low temperatures), toxic heavy metal stress, as well as different biotic stresses (see [14] and references therein).

Although most bacteria can generate and sense H_2_S, the exact physiological roles of this compound in the prokaryotes are not yet wholly understood. In particular, whether endogenous H_2_S is a true signaling effector molecule that induces a response in the same microbial cell, or whether the H_2_S-mediated effect is more a response to environmental and/or host-derived H_2_S, remains to be established [6]. Meanwhile, Shatalin et al. reported that inhibition of H_2_S production through inactivation of H_2_S-generating enzymes in *Bacillus anthracis* Sterne, *Pseudomonas aeruginosa* PA14, *Staphylococcus aureus* (MSSA RN4220 and MRSA MW2), and *Escherichia coli* MG1655 makes these pathogenic species very sensitive to different classes of antibiotics [15]. The addition of exogenous H_2_S reverses this effect. The authors concluded that endogenously generated H_2_S increases bacterial resistance to oxidative stress imposed by antibiotics. The antibiotic-induced ROS cause DNA damage via the Fenton reaction. It is suggested that H_2_S prevents oxidative DNA damage in bacteria via the following cytoprotective mechanisms: (i) direct reduction of H_2_O_2_ into H_2_O; (ii) depletion of Fe^2+^, a catalyst of the Fenton reaction; (iii) transient depletion of free cysteine, a reducing agent that fuels the Fenton reaction; and (iv) stimulation of the activities of superoxide dismutase (SOD) and catalase [15]. The latter two enzymes are the well-known ROS scavenging systems [16]. It should be noted, however, that Shatalin et al. [15] did not suggest a mechanism for the depletion of Fe^2+^ and free cysteine by H_2_S.

H_2_S is a colorless flammable gas, soluble in water (100 mM at 25 °C [17]), and with high cell membrane permeability [18]. It is a weak acid; therefore in aqueous solutions, it equilibrates with hydrosulfide (HS^−^) and sulfide (S^2−^) according to Equation (1):

(1) $$H_{2}S\overset{pK_{a1}}{\Leftrightarrow }{\mathrm{HS}}^{-}+H^{+}\overset{pK_{a2}}{\Leftrightarrow }S^{2-}+2H^{+}$$

According to the reported p*K*_a1_ values that varied from 6.97 to 7.06 at 25 °C (see [19] and references therein), at physiological pH 7.4, 69–73% of the total hydrogen sulfide pool in the solution is in the form of HS^−^, and 27–31% exists as H_2_S. Taking into account the reported p*K*_a2_ values (from 12.2 to 19), the concentration of S^2−^ in the solution at pH 7.4 is negligible. The mitochondrial matrix in eukaryotic cells is usually alkaline, with a pH value of about 8 [20,21]. Accordingly, in the matrix, the concentration of HS^−^ is higher, 90–91%, and the remainder is in the form of H_2_S. On the contrary, intralysosomal pH in living cells is highly acidic, 4.7–4.8 [22]. Therefore, inside lysosomes, the undissociated form of hydrogen sulfide, H_2_S, dominates (>99%). H_2_S acts as a reducing agent. Respectively, the standard redox potential (vs. the standard hydrogen electrode, pH 7) *E*^0^′(S^0^/H_2_S) is −230 mV (*E*^0^′(S^0^/HS^−^) = −270 mV) [19]. Herein, unless otherwise stated, we use the term “H_2_S” to designate the total hydrogen sulfide pool (H_2_S + HS^−^ + S^2−^).

This review focuses on the effects of H_2_S on the respiratory chains of mammalian mitochondria and bacteria, mammalian F_o_F_1_-ATP synthase, and mammalian lactate dehydrogenase (LDH) in light of recent findings.

## 2. Endogenous Production of H_2_S

In mammalian tissues, H_2_S can be endogenously produced via both non-enzymatic and enzymatic pathways.

### 2.1. Non-Enzymatic Production of H_2_S

**Figure 2:**
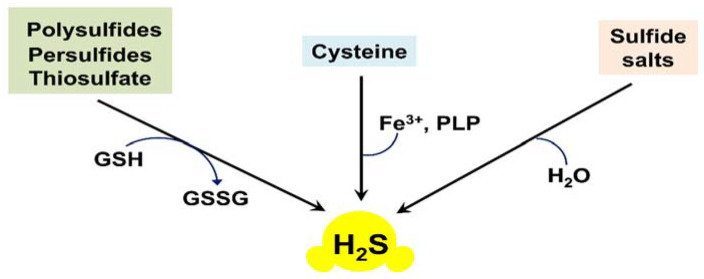
Non-enzymatic endogenous production of H_2_S in mammalian tissues.

Non-enzymatic formation of H_2_S usually takes place in the reactions of thiols or thiol derivatives with other molecules [12,13,23]. Inorganic polysulfides, persulfides, and thiosulfate can be reduced with reduced glutathione (GSH) to yield H_2_S (Figure 2). This demands the presence of reducing equivalents, such as NADPH (reduced nicotinamide adenine dinucleotide phosphate), because glutathione disulfide (GSSG), also produced in the reaction, needs to be converted back to GSH by NADPH-dependent glutathione reductase. Inorganic sulfide salts, such as Na_2_S or NaHS, can undergo hydrolysis. Yang et al. also reported that cysteine is the preferred substrate for the non-enzymatic pathway, and that the reaction required coordinated catalysis by Fe^3+^ and pyridoxal phosphate (PLP) [24] (Figure 2).

### 2.2. Enzymatic Production of H_2_S

**Figure 3:**
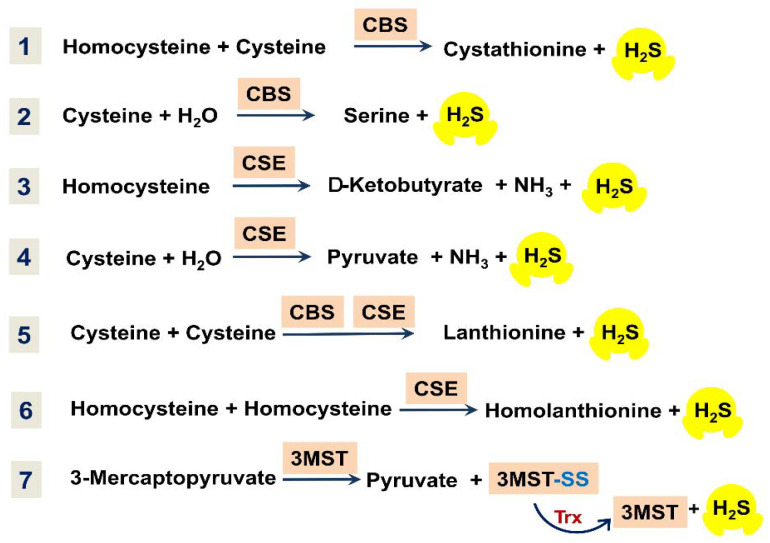
Overview of main reactions for enzymatic production of H_2_S in mammalian tissues.

Enzymatic production of H_2_S in mammalian systems is carried out primarily by cystathionine-β-synthase (CBS), cystathionine-γ-lyase (CSE), and 3-mercaptopyruvate-sulfurtransferase (3MST) [12,13,23,25,26]. The main enzymatic reactions resulting in the biosynthesis of H_2_S are shown in Figure 3. CBS can catalyze the condensation of homocysteine and L-cysteine to produce L-cystathionine and H_2_S (Figure 3, reaction 1). In the presence of L-cysteine, CBS can also produce H_2_S, with the formation of L-serine as a byproduct (Figure 3, reaction 2). CSE can decompose homocysteine to yield H_2_S, α-ketobutyrate, and ammonia (Figure 3, reaction 3). Furthermore, CSE can catalyze the conversion of L-cysteine into H_2_S, pyruvate, and ammonia (Figure 3, reaction 4). Both CBS and CSE can generate H_2_S through β-replacement of cysteine by a second cysteine, with the formation of lanthionine as a byproduct (Figure 3, reaction 5). Additionally, CSE can catalyze the γ-replacement reaction between two homocysteine molecules, with the production of H_2_S and homolanthionine as a by-product (Figure 3, reaction 6). 3MST generates H_2_S from 3-mercaptopyruvate coupled with either of the two enzymes, mitochondrial cysteine aminotransferase (CAT) or peroxisomal D-amino acid oxidase (DAO). 3MST transfers a sulfur atom from 3-mercaptopyruvate onto itself, resulting in the formation of the enzyme-bound persulfide (3MST-SS) and pyruvate (Figure 3, reaction 7). H_2_S is then released from the persulfide in the presence of a reductant, e.g., thioredoxin (Trx). CAT and DAO in turn produce 3-mercaptopyruvate from L-cysteine and D-cysteine, respectively [13].

The majority of bacterial species whose genomes were completely sequenced have the orthologs of mammalian genes encoding CBS, CSE, or 3MST [15]. Since H_2_S provides defense against modern antibiotics in bacteria, suppression of H_2_S-producing enzymes in pathogens by new drugs would be a promising antimicrobial treatment strategy [15,27]. The use of H_2_S biogenesis as a target for versatile antibiotic potentiators may have therapeutic potential for the fight against difficult-to-treat infections based on bacterial antibiotic tolerance.

## 3. *S*-Sulfhydration of ATP Synthase

In eukaryotes, one of the main targets of H_2_S signaling is the mitochondria. These cell organelles are known to be the power plants of the eukaryotic cell. Energy transduction events in mitochondria occur in the O_2_-dependent respiratory electron transport chain. The mammalian respiratory chain is unbranched [28,29]. It consists of four membrane-bound multi-subunit complexes: I, (reduced nicotinamide adenine dinucleotide) NADH: ubiquinone reductase or type I NADH dehydrogenase; II, succinate dehydrogenase; III, ubiquinol: cytochrome *c* reductase or cytochrome *bc*_1_ complex; and IV, cytochrome *c* oxidase or cytochrome *aa*_3_. The chain transfers electrons from NADH and succinate to O_2_. The electron transfer reactions catalyzed by complexes I, III, and IV are coupled to the generation of the proton motive force. The latter is used by ATP synthase (F_o_F_1_-ATP synthase or complex V) to produce ATP.

**Figure 4:**
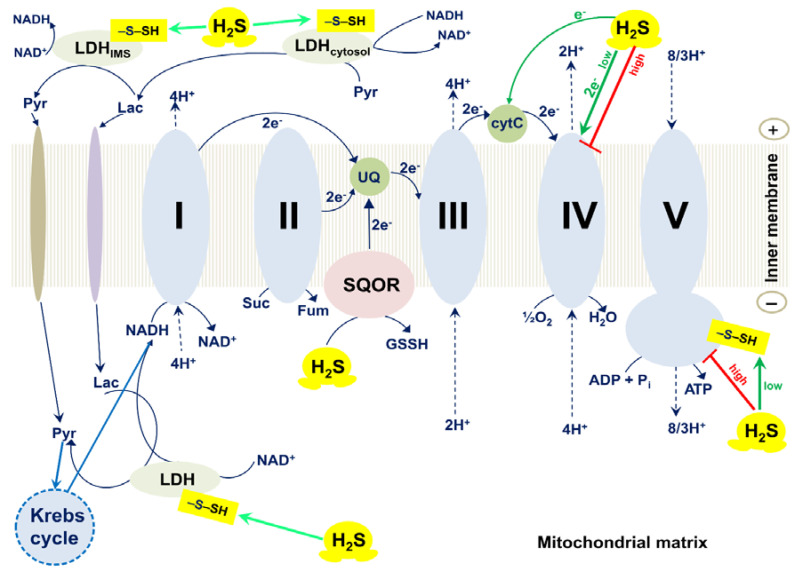
Effects of H_2_S on mammalian mitochondrial electron transport chain, ATP synthase, and lactate dehydrogenase. The mammalian respiratory chain includes four different membrane-bound complexes: I, II, III, and IV. H_2_S at low concentrations stimulates the activity of F_o_F_1_-ATP synthase (also known as complex V) via *S*-sulfhydration of Cys244 or Cys294 of its α subunit [30]. H_2_S increases the activity of lactate dehydrogenase (LDH) via *S*-sulfhydration of its Cys163 that, in turn, stimulates mitochondrial electron transport [31]. H_2_S can also donate electrons to the respiratory chain via sulfide quinone oxidoreductase (SQOR) by directly reducing ubiquinone (UQ) [32]. At high (toxic) concentrations H_2_S inhibits complex IV (cytochrome *c* oxidase) and F_o_F_1_-ATP synthase that leads to reversible inhibition of mitochondrial electron transport and ATP production [30,33,34,35]. At low concentrations H_2_S serves as an electron donor for complex IV, either directly [36,37,38] or indirectly, via reduction of its native substrate cytochrome *c* [39]. The outer mitochondrial membrane is not shown for simplicity.

Modis et al. observed *S*-sulfhydration of subunit α of the synthase (ATP5A1) in HepG2 and HEK293 cell lysates in response to exposure to H_2_S, using a biotin switch assay [30]. Sulfhydration of subunit α of the synthase increases with increasing the H_2_S concentration, 50–300 μM. H_2_S at low concentrations (10–100 nM) stimulates the specific activity of ATP synthase, while at higher concentrations (1–10 μM) a tendency for inhibition of the activity is detected (Figure 4). Such a bell-shaped concentration–response curve is quite typical for the effects of H_2_S. Sulfhydration occurs at two highly conserved cysteine residues in subunit α of the synthase, Cys244, or Cys294. Mutation of either of the two cysteines (C244S or C294S) leads to a slight reduction in the catalytic activity of ATP synthase. The double mutant (C244S/C294S) exhibits more than 50% inhibition of the enzyme activity. In vivo, subunit α of the synthase is basally sulfhydrated. The basal sulfhydration is mostly due to CSE-derived endogenous H_2_S generation because it is suppressed in liver homogenates harvested from CSE^−/−^ mice. Burn injuries that upregulate CSE and increase H_2_S generation result in an increase in *S*-sulfhydration of subunit α of the synthase. Thus, *S*-sulfhydration of subunit α of the synthase could be a physiological mechanism to maintain ATP synthase in a physiologically activated state, thereby supporting mitochondrial bioenergetics [30].

## 4. *S*-Sulfhydration of Lactate Dehydrogenase (LDH)

LDH catalyzes the reversible conversion of lactate to pyruvate with the reduction of NAD^+^ to NADH, and vice versa. The enzyme is a tetramer that is usually composed of the two most common types of subunits, LDHA and LDHB [40]. LDHA and LDHB can assemble into five different isoenzymes: LDH1, LDH2, LDH3, LDH4, and LDH5. Isoenzymes that are rich in LDHA catabolize pyruvate to lactate with the concomitant production of NAD^+^ from NADH. Conversely, isoenzymes rich in LDHB facilitate lactate-to-pyruvate conversion with the concomitant formation of NADH from NAD^+^.

Untereiner et al. reported that in the colon cancer cell line HCT116, LDHA catalyzes the conversion of pyruvate to lactate, and H_2_S donation increases the total cellular lactate levels [31]. Notably, H_2_S enhances the catalytic activity of LDHA, which is primary cytosolic, doing so via *S*-sulfhydration of its Cys163 (Figure 4). Experiments with whole HCT116 cell extracts showed that H_2_S also stimulates the LDHB activity, although to a smaller extent. Importantly, H_2_S stimulates oxidative phosphorylation in HCT116 cells in an LDHA-dependent manner. Thus, in colon cancer cells, H_2_S-induced stimulation of the catalytic activity of LDHA leads to the stimulation of mitochondrial electron transport. The authors hypothesize that the increase in the LDHA activity causes an increase in cytosolic lactate. This enhances the flux of lactate into the mitochondria through the intracellular lactate shuttle. Lactate enters the mitochondrial intermembrane space. Lactate and pyruvate also enter the mitochondrial matrix. Lactate is converted to pyruvate via the mitochondrial LDH that is primarily made up of LDHB and also activated by H_2_S. Pyruvate in the matrix is oxidized via the Krebs cycle to generate NADH that stimulates the activity of the mitochondrial respiratory chain. Additionally, the oxidation of lactate to pyruvate by the mitochondrial LDH is coupled to the reduction of NAD^+^ to NADH. This NADH, in turn, is utilized by the mitochondrial respiratory chain to further support electron transport.

## 5. H_2_S Donates Electrons to the Mitochondrial Respiratory Chain via Sulfide Quinone Oxidoreductase (SQOR)

Powell and Somero first reported that the oxidation of H_2_S can occur in mitochondria, and that the process is coupled to oxidative phosphorylation [41]. The mitochondrial oxidation of H_2_S was observed in the gill and foot tissue of *Solemya reidi*, a gutless clam living in sulfide-rich habitats. Later, Goubern et al., using permeabilized human colon adenocarcinoma HT29 cells, showed that H_2_S at low micromolar concentrations can serve as an electron donor for the mammalian respiratory chain [32]. This is accompanied by mitochondrial energization. The latter is extremely sensitive to the amount of H_2_S delivered instantaneously to mitochondria. H_2_S donates electrons at the respiratory chain at the level of UQ.

The enzyme that catalyzes the electron transfer from H_2_S to UQ is SQOR [42]. SQOR belongs to group four of the flavin disulfide reductase (FDR) superfamily. The enzyme contains a tightly bound FAD (flavin adenine dinucleotide) and two spatially proximal redox-active cysteines. A peculiar feature of human SQOR is the fact that the two cysteines are linked via a bridging sulfur atom which forms the redox-active cysteine trisulfide configuration. The trisulfide appears to be the active form of SQOR, being present at the start and end of the catalytic cycle [43].

When H_2_S is oxidized in the mitochondrial matrix by the action of SQOR under physiological conditions, GSH serves as the primary sulfur acceptor (Figure 4). In the reaction that is coupled to the reduction of UQ to UQH_2_, glutathione persulfide (GSSH) is produced. GSSH is then oxidized by persulfide dioxygenase (ETHE1) to yield sulfite (SO_3_^2−^) and regenerate GSH. GSSH can also be a substrate for rhodanese or thiosulfate sulfurtransferase (TST). TST catalyzes the reaction of GSSH with SO_3_^2−^ to generate GSH and thiosulfate (S_2_O_3_^2−^). Alternatively, SO_3_^2−^ can be oxidized to sulfate (SO_4_^2−^) by sulfite oxidase (SUOX) located in the intermembrane space, with the concomitant reduction of cytochrome *c*. Thiosulfate and sulfate can be excreted with urine (see [42] and references therein). Thus, electrons from the sulfide oxidation pathway can enter the mitochondrial respiratory chain at the level of the cytochrome *bc*_1_ complex (from SQOR) and cytochrome *c* oxidase (from SUOX).

## 6. H_2_S at Toxic Concentrations Inhibits Mitochondrial Cytochrome *c* Oxidase

H_2_S exposure, at high concentrations and high rates, is extremely toxic to mammals, including humans [35]. The acute toxicity of H_2_S is generally attributed to the suppression of the mitochondrial cytochrome *c* oxidase (complex IV). Complex IV couples the transfer of electrons from cytochrome *c* to O_2_ with the generation of the transmembrane proton gradient using the mechanism of proton pumping [16,44,45,46,47,48,49,50,51,52,53,54,55,56,57,58,59,60,61,62,63,64,65]. The enzyme carries four redox-active metal sites: Cu_A_, heme *a*, heme *a*_3_, and Cu_B_. Electrons from cytochrome *c* primarily accepted by Cu_A_ are transferred to heme *a* and then to the catalytic binuclear center composed of heme *a*_3_ and Cu_B_ in which the reduction of oxygen to water occurs [66,67,68,69]. There are two forms of cytochrome *c* oxidase: slow (resting oxidase as obtained from the preparation) and fast (the completely reduced enzyme exposed to a “pulse” of O_2_) [70]. The fast form is also called pulsed or unrelaxed. The slow form differs from the fast form in lower reactivity for inhibitors [71] and slower intramolecular electron transfer [72].

H_2_S at high concentrations was reported to inhibit the O_2_ consumption by the beef heart cytochrome *c* oxidase in the isolated form and in submitochondrial particles (Figure 4) [33,34,73]. The inhibition by H_2_S appears to be non-competitive with respect to both substrates, cytochrome *c* and O_2_ [34]. The inhibition efficiency increases with decreasing pH: the *K*_i_ values measured at pH 8.05, 7.48, and 6.28, are 2.6, 0.55, and 0.07 μM, respectively [37]. The initial rate of inactivation of the fast form of the isolated cytochrome *c* oxidase is proportional to total H_2_S concentration, with an initial rate constant, *k*_on_, of 2.2 × 10^4^ M^−1^ s^−1^ at pH 7.47 [38]. The inhibition of complex IV leads to blockage of the mitochondrial respiratory chain and, as a consequence, to the dissipation of the membrane potential, the cessation of oxidative phosphorylation, and enhanced ROS production [35]. Consistently, examination of the cytotoxic effects of inhalational H_2_S exposure showed that sublethal (>50 ppm) concentrations of the inhaled gas caused inhibition of cytochrome *c* oxidase activity in the lung and heart, which can be observed ex vivo and is associated with pathological alterations [74,75,76,77,78,79]. That complex IV is suppressed by high (usually 10–100 μM) concentrations of H_2_S in vitro in various cell lines is well documented [75,80,81,82,83,84,85]. The H_2_S-induced inhibition is reversible, e.g., 10–30 min after the exposure of tissue homogenates to H_2_S, the activity of complex IV gets back to its initial level, simultaneously with the decomposition of the inhibitor [86].

**Figure 5:**
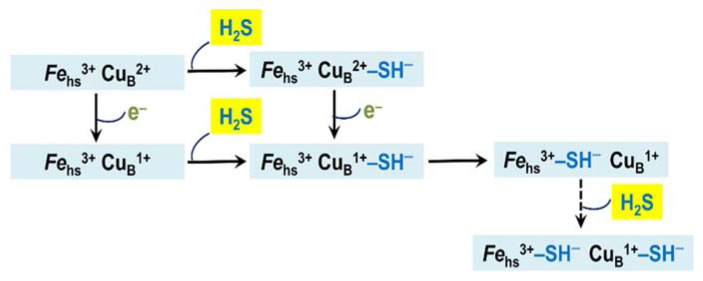
Proposed mechanism of inhibition of mitochondrial cytochrome *c* oxidase and *Escherichia coli* cytochrome *bo*_3_ by H_2_S. Shown is the catalytic binuclear center in different redox and ligation states. The center consists of the copper ion Cu_B_ and the high-spin heme (*Fe*_hs_). The latter is heme *a*_3_ in cytochrome *c* oxidase and heme *o*_3_ in cytochrome *bo*_3_. The metal redox groups which are not part of the center are not shown.

H_2_S, presumably in the form of HS^−^, binds to the ferric heme *a*_3_ and Cu_B_ in either state (either cupric or cuprous) [38]. The final enzyme product of H_2_S inhibition is a mixed-valence state of the oxidase in which Cu_A_ and heme *a* are reduced and heme *a*_3_ is in the ferric H_2_S-bound form [33,87]. Cu_B_ in the final inhibited enzyme remains reduced even after reoxidation of the H_2_S-bound oxidase, as evidenced by an electron paramagnetic resonance spectroscopy study [88]. The fact that Cu_B_ remains cuprous in the presence of ferricyanide suggests that H_2_S binding to Cu_B_^1+^ increases its redox potential and makes re-oxidation difficult. In other words, Cu_B_^1+^ in this state may also be H_2_S-bound [88]. Nicholls et al. [38] suggested a model for the H_2_S-mediated inhibition of the mitochondrial cytochrome *c* oxidase (Figure 5). According to this model, during the steady-state turnover of complex IV, the first molecule of HS^−^ transiently binds to cupric or cuprous Cu_B_. Then, it is transferred to the ferric heme *a*_3_ that blocks the catalytic reaction of the heme with O_2_. In the final protein-inhibitor adduct, Cu_B_ is likely reduced and presumably bound to the second molecule of HS^−^. Heme *a* and Cu_A_ are most likely reduced in the adduct.

## 7. H_2_S at Low Concentrations Serves as Electron Donor for Mitochondrial Cytochrome *c* Oxidase

**Figure 6:**
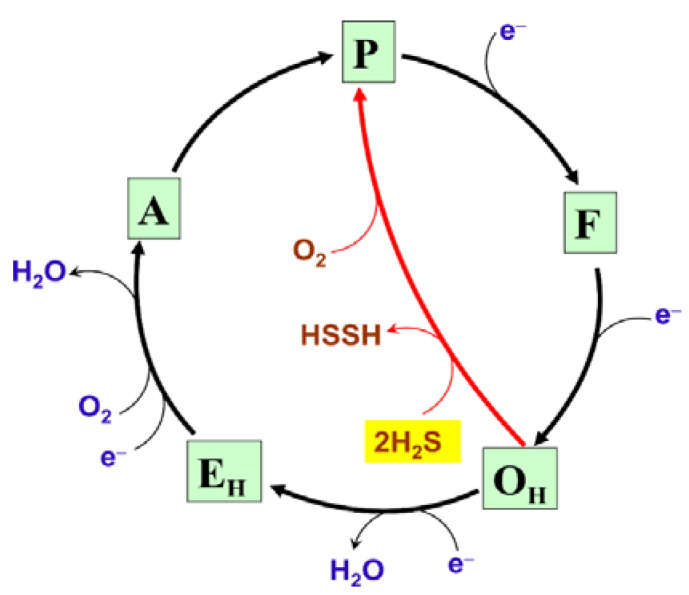
Proposed molecular mechanism of the reaction of the fast form of mitochondrial cytochrome *c* oxidase with low H_2_S levels. O_H_, E_H_, A, P, and F are unrelaxed oxidized, unrelaxed one-electron-reduced, compound ‘A’, compound ‘P’, and compound ‘F’ catalytic intermediates, respectively. Chemical and pumped protons involved in the catalytic cycle are not shown for clarity.

H_2_S at low concentrations can act as a substrate for mitochondrial complex IV (Figure 4) [36,37]. The fast form of the oxidized cytochrome *c* oxidase reacts aerobically with low H_2_S levels in the absence of reduced substrates. However, the initial product of this reaction is not the inhibited enzyme but a catalytic intermediate, compound ‘P’ (Figure 6) [38]. Compound ‘P’ then decays into another catalytic intermediate, compound ‘F’. It is proposed that in this reaction two H_2_S molecules donate two electrons to the fully oxidized binuclear center, *a*_3_^3+^Cu_B_^2+^, converting it to the fully reduced state, *a*_3_^2+^Cu_B_^+^. The concomitant H_2_S oxidation product is possibly hydrogen persulfide (HSSH), although a form of free sulfur (S^0^ or S_8_) cannot be excluded [38]. Then the fully reduced binuclear center reacts with O_2_, producing compound ‘P’. It remains unclear whether cytochrome *c* oxidase contributes significantly to a dissimilatory mechanism for the endogenously generated H_2_S, or if SQOR is the major contributor in vivo. Notably, H_2_S can also reduce complex IV indirectly, via reduction of its native substrate cytochrome *c* (Figure 4). The reaction between H_2_S and cytochrome *c* presumably leads to the initial formation of the HS^•^/S^•−^ radical. HS^•^/S^•−^ could then be trapped by proteins producing protein persulfides and superoxide, or be trapped by O_2_ yielding HSO_2_^•^ [39].

## 8. Effect of H_2_S on the Operation of the Branched Respiratory Chain of *E. coli* and Bacterial Growth

Under steady-state conditions, the mammalian tissue concentration of H_2_S is usually in the low nanomolar range, e.g., it is around 15 nM in mouse brain and liver tissues [89]. The mammalian gut, in this sense, is unique among other body compartments. Millimolar concentrations (1.0–2.4 mM) of H_2_S are commonly present in the gut [90]. The reason is that in the gastrointestinal tract, unlike other compartments, H_2_S is generated by both the mammalian CBS, CSE, and 3MST, and by the microbial communities inhabiting the gut, of which sulfate-reducing bacteria are the key H_2_S-producing species [90,91]. At such extremely high H_2_S levels, bacterial respiratory chains which terminate in cytochrome *c* oxidase or other heme-copper oxidases should be inhibited. The question then arises as to how under these conditions bacteria inhabiting the mammalian gut can maintain aerobic respiration. It turned out that *E. coli*, and possibly other bacteria inhabiting H_2_S-rich environments, has a unique *bd*-type terminal oxidase that is not inhibited by H_2_S, even at toxic concentrations [92,93,94,95].

**Figure 7:**
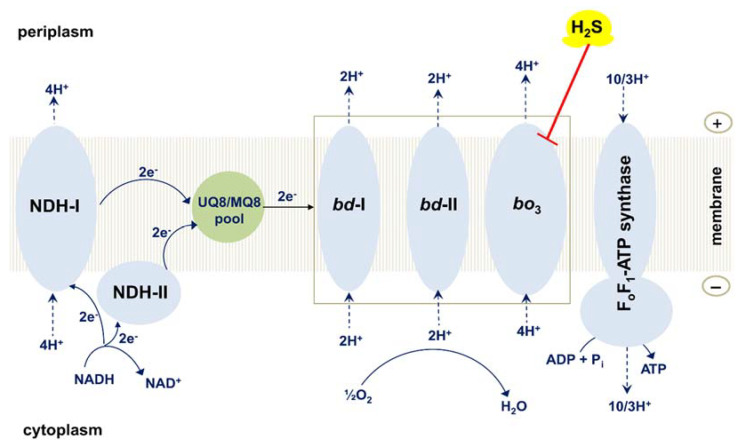
Effect of H_2_S on the operation of the branched respiratory chain of *E. coli*. Shown is the schematic view of the branched aerobic respiratory chain of *E. coli*. H_2_S inhibits cytochrome *bo*_3_ but does not affect cytochrome *bd*-I and cytochrome *bd*-II. Succinate dehydrogenase and other substrate dehydrogenases are not shown for clarity.

*E. coli* is an essential member of the intestinal microbiome of humans and warm-blooded animals. The large intestine of humans normally harbors several *E. coli* strains at a given point in time [96]. In contrast to the electron transport chain of mammals that is unbranched, *E. coli*, like many other prokaryotes, possesses the branched aerobic respiratory chain (Figure 7) [45,97,98,99,100,101,102]. The *E. coli* chain comprises of type I and type II NADH dehydrogenases which transfer electrons from NADH to ubiquinol-8 (UQ8) or menaquinol-8 (MQ8); succinate dehydrogenase transferring electrons from succinate to UQ8; and three terminal oxidases, cytochromes *bo*_3_, *bd*-I, and *bd*-II which transfer electrons from UQ8 or MQ8 to O_2_ producing H_2_O [103,104,105,106,107,108,109,110,111,112]. The proton motive force generated by type I NADH dehydrogenase and the terminal oxidases is used by F_o_F_1_-ATP synthase to make ATP [113,114,115]. Being a true proton pump, cytochrome *bo*_3_ produces the proton motive force with higher efficiency as compared to the evolutionarily unrelated *bd*-type oxidases [116,117,118,119,120,121]. The three-dimensional structures of cytochrome *bo*_3_ and cytochrome *bd*-I were determined [122,123,124,125]. The *bo*_3_ oxidase is a member of type A-1 heme-copper oxidase superfamily [46,48,49,51,54,56,57,58,59,61,62,63]. Cytochrome *bo*_3_ contains the UQ8 binding site, heme *b*, and the catalytic binuclear center formed by heme *o*_3_ and Cu_B_ [101,126]. Cytochrome *bd*-I and cytochrome *bd*-II are members of the L subfamily of the cytochrome *bd* oxygen reductase family [127,128]. According to recent work by Murali et al. [129], the latter family should be expanded into a huge superfamily. The *bd* oxidase has the UQ8/MQ8 binding site, and three heme prosthetic groups, *b*_558_, *b*_595_, and *d* but lacks any copper site [97,98,108,127,130,131,132,133]. Hemes *b*_595_ and *d* may form a di-heme active site for oxygen chemistry, as evidenced by a number of studies [134,135,136,137,138,139,140,141,142,143,144,145,146,147]. These hemes are in van der Waals contacts [124,125], enabling fast electron transfers between them [119,145,146]. Thus, the functional di-heme active site in the *bd* oxidase implies that heme *b*_595_ is able to rapidly donate an electron and a proton to heme *d* to perform a concerted four-electron reduction of oxygen to water. This role of heme *b*_595_ in cytochrome *bd* is similar to that of Cu_B_ in the catalytic binuclear center of cytochrome *bo*_3_. Cytochromes *bo*_3_ and *bd* have low and high O_2_ affinity, respectively [148,149,150,151,152,153]. As a consequence, the *bo*_3_ enzyme dominates in *E. coli* under conditions of high O_2_ concentration, whereas the *bd* oxidase is expressed at low aeration [154,155,156,157,158]. Cytochrome *bd* performs vital physiological functions in *E. coli* and other prokaryotes [16,131,159,160,161,162,163]. In particular, the enzyme endows the microbes with resistance to nitric oxide [164,165,166,167,168,169,170,171,172,173], peroxynitrite [160,174], hydrogen peroxide [16,175,176,177,178,179,180], cyanide [92,181,182], and ammonia [183]. The *bd*-type oxidases are present in the respiratory chains of bacteria and archaea but not in humans and animals. For this reason, they could serve as suitable protein targets for next-generation antibiotics [133,171,184,185,186,187,188,189,190].

Forte et al. studied the effect of H_2_S on the oxygen consumption by the purified terminal oxidases *bo*_3_, *bd*-I, and *bd*-II from *E. coli* [92]. It turned out that the activity of cytochrome *bo*_3_ is quickly inhibited with an apparent half-maximal inhibitory concentration *IC*_50_ of 1.1 μM H_2_S (pH 7.4). This value is similar to the *K*_i_ of 0.55 μM H_2_S obtained by Nicholls and Kim for the beef heart cytochrome *c* oxidase at pH 7.48 [37]. The inhibition of the *bo*_3_ oxidase appeared to be fully reversible. The rapid and total recovery of the enzymatic activity occurs following the removal of H_2_S from the solution by the H_2_S scavenger, the *Entamoeba histolytica O*-acetylserine sulfhydrylase (*Eh*OASS), in the presence of excess *O*-acetyl-L-serine (OAS). In contrast, neither the *bd*-I oxidase nor the *bd*-II oxidase is inhibited under identical conditions, even at a high concentration of H_2_S (58 μM) [92]. Similar results were obtained when examining the effect of H_2_S on the oxygen consumption by cell suspensions of the *E. coli* mutant strains which possess *bo*_3_, *bd*-I, and *bd*-II as the only terminal oxidase. The oxygen uptake by cells having *bo*_3_ as the sole oxidase is rapidly inhibited by 50 µM H_2_S. As in the case of the purified enzyme, the inhibition is promptly and completely restored after H_2_S depletion by the *Eh*OASS/OAS system. Conversely, H_2_S at the same concentration does not affect the oxygen uptake by mutant cells having either *bd*-I or *bd*-II as the only oxidase [92]. Similar data on the *E. coli* membrane vesicles were reported by Korshunov et al. [93].

Forte et al. also tested if *bd*-I and/or *bd*-II oxidases, besides enabling aerobic respiration, promote the growth of *E. coli* cells in the presence of H_2_S [92]. A quantity of 200 μM H_2_S appeared to severely inhibit the growth of mutant cells with only cytochrome *bo*_3_. On the contrary, virtually no effect on cell growth was detected following the addition of H_2_S at the same concentration to the strains which express either *bd*-I or *bd*-II as the sole oxidase. Thus, in contrast with cytochrome *bo*_3_, which is potently and reversibly inhibited by H_2_S, both cytochrome *bd*-I and cytochrome *bd*-II are H_2_S-insensitive, and therefore, able to sustain respiration and cell growth in the presence of high levels of H_2_S. We assume that the mechanism of the inhibition of the *E. coli* cytochrome *bo*_3_ by H_2_S is very similar to that suggested for the H_2_S-mediated inhibition of another heme-copper terminal oxidase, mitochondrial complex IV (Figure 5).

The H_2_S resistance of cytochrome *bd* is probably a key trait not only found in *E. coli*. Saini et al. [191] reported that H_2_S promotes the respiration and growth of *Mycobacterium tuberculosis*, *Mycobacterium smegmatis*, and *Mycobacterium bovis* BCG. The authors concluded that the suppression of the cytochrome *bcc*-*aa*_3_ supercomplex by H_2_S leads to the switching of the electron flow from MQ to cytochrome *bd*. The latter, in turn, stimulates respiration and ATP production, leading to the increased growth of the mycobacteria [191]. Furthermore, Kunota et al. [192] showed that multidrug-resistant and drug-susceptible clinical *M. tuberculosis* strains (but not non-pathogenic *M. smegmatis*) generate H_2_S endogenously, maintaining bioenergetic homeostasis by stimulating respiration primarily via the *bd*-type terminal oxidase. These findings are in agreement with the fact that the *bd* oxidases of the *E. coli* respiratory chain are H_2_S-insensitive [92,93].

## 9. Concluding Remarks

Accumulated evidence has shown that H_2_S is an effector molecule that controls energy metabolism. Depending on the concentration, H_2_S can either stimulate or inhibit the mammalian mitochondrial respiratory chain and F_o_F_1_-ATP synthase. An involvement of LDHA in the stimulatory effects of H_2_S on mitochondrial respiration has also been suggested. High H_2_S inhibits the bacterial respiratory chain terminating in cytochrome *bo*_3_ but not cytochrome *bd*. The effects of H_2_S on a bacterial ATP synthase are still unknown. Although the role of H_2_S in microorganisms has been investigated less, there is currently an explosion of interest in the link between H_2_S and bacterial energy metabolism as a result of the recognition that this gaseous signaling molecule is critically important for the growth and colonization potential of pathogens, such as *M. tuberculosis.* A clearer picture of the effects of H_2_S on energy metabolism would represent a significant advance for the development of novel therapeutics.
